# Antibiotic Therapy and Factors Predicting Prolonged Hospitalization Among Children Under Three Years Diagnosed With Respiratory Syncytial Virus‐Associated Lower Respiratory Infection

**DOI:** 10.1002/iid3.70370

**Published:** 2026-02-16

**Authors:** Ali Alsuheel Asseri

**Affiliations:** ^1^ Department of Child Health, College of Medicine King Khalid University Abha Saudi Arabia

**Keywords:** antibiotics, bronchiolitis, hospitalization, respiratory syncytial virus, Saudi Arabia

## Abstract

**Background:**

Respiratory syncytial virus (RSV) is a common cause of lower respiratory infections (LRIs) in infants and young children. Antibiotic overuse remains a significant concern in hospitalized children with RSV‐associated LRIs. This study aimed to investigate the prevalence of antibiotic use and identify predictors of prolonged hospitalization in children with RSV‐LRIs.

**Methods:**

A retrospective record review study was conducted at Abha Maternity and Children's Hospital, enrolling 162 children aged 1–36 months admitted with RSV‐associated LRIs between January and December 2022. Demographic, clinical, laboratory, and imaging data were collected. Antibiotic therapy and hospital length of stay (LOS) were also retrieved and analyzed.

**Results:**

Of the 162 patients, 147 (90.74%) received antibiotic therapy, with azithromycin, cefuroxime, and ceftriaxone being the most commonly used. Patients who received antibiotics had a significantly longer median LOS compared to those who did not (6 vs. 3 days, *p* < 0.001). Factors associated with prolonged LOS (≥ 5 days) included antibiotic therapy use (odds ratio [OR] = 7.47 (95% confidence intervals [CI]: 2.18−25.57), pneumonia: OR = 3.58 (95% CI: 1.67−7.67), age < 12 months: OR = 2.80 (95% CI: 1.32−5.89), consolidation: OR = 2.54 (95% CI: 1.16−5.59), age in months: OR = 0.94 (95% CI: 0.91−0.98) (indicates decreasing odds with increasing age), admission respiratory rate: OR = 1.04 (95% CI: 1.01−1.07), and admission peripheral oxygen saturation (SpO_2_): OR = 0.91 (95% CI: 0.86−0.97) (indicates decreasing odds with higher SpO_2_).

**Conclusion:**

The high prevalence of antibiotic use in this study highlights the challenges in differentiating RSV‐LRIs from bacterial pneumonia. Independent predictors of prolonged hospital stay included antibiotic therapy, pneumonia, younger age, lower admission SpO₂, and higher respiratory rate.

## 1 Introduction

Respiratory syncytial virus (RSV) is a highly contagious, single‐stranded RNA pneumovirus that causes lower respiratory infections (LRIs) predominantly affecting infants and young children [1, 2, 3, 4]. Within this age group, it is the most common cause of bronchiolitis, a lower respiratory illness characterized by inflammation of the small airways [1, 5]; in fact, RSV causes more than 80% of LRIs among pediatric populations worldwide. Bronchiolitis is clinically characterized by prodromal symptoms and shortly progresses to shortness of breath and hypoxia. While most RSV infections resolve spontaneously within a week [6], some children, particularly those under 2 years old and those with cardiopulmonary comorbidities, experience severe symptoms requiring hospitalization [1, 2, 7, 8, 9]. Consequently, RSV infection in infants is characterized by significant morbidity and mortality rates and is associated with a substantial healthcare burden [3, 10, 11, 12, 13, 14].

Despite RSV being a viral infection for which antibiotics are ineffective, some studies have reported concerningly high rates of antibiotic use among hospitalized children with RSV‐LRI [15, 16]. This inappropriate use is a major public health concern, as overuse of antibiotics contributes to the development of antibiotic resistance, a global threat that poses significant challenges in treating bacterial infections [15, 17, 18]. Additionally, unnecessary antibiotic use can expose children to potential side effects ranging from mild nausea and diarrhea to more serious allergic reactions. Furthermore, it increases healthcare costs due to the additional expense of the medications and the potential for extended hospital stays [15, 19].

Understanding the factors associated with prolonged hospitalization in children with RSV‐LRIs can be a valuable tool in guiding more judicious antibiotic prescribing practices. By identifying these factors, clinicians can better distinguish between children who are likely to experience a more severe illness and may benefit from additional interventions, and those who are likely to recover well with supportive care alone. This targeted approach can help reduce unnecessary antibiotic use while ensuring optimal patient outcomes [20]. The present study aims to investigate the prevalence of antibiotic use and identify potential predictors of prolonged hospitalization (≥ 5 days) among children under 3 years old who are hospitalized with RSV‐LRI.

## 2 Materials and Methods

### 2.1 Study Population and Data Extraction

A retrospective record review study was conducted at Abha Maternity and Children Hospital, enrolling 162 children aged 1–36 months admitted between January and December 2022 with laboratory‐confirmed RSV‐associated LRI. LRI was defined as per the WHO criteria [1], requiring the presence of fever (≥ 38°C), cough, and shortness of breath within the preceding 10 days. All participants underwent Reverse Transcription Polymerase Chain Reaction testing for RSV confirmation and received pediatric ward hospitalization. Critically ill patients requiring direct critical care admission were excluded. Demographic, clinical, laboratory, and imaging data, antibiotic therapy, and hospital outcome measures were collected. Clinical findings included presenting symptoms and signs as well as coexisting medical conditions. Prematurity was defined as a birth before 37 weeks’ gestational age, while chronic lung disease of prematurity, also known as bronchopulmonary dysplasia, was defined as presence of prematurity with oxygen requirement for more than 4 weeks postdelivery [21, 22]. Presence of congenital heart disease was also documented based on available echocardiogram findings (yes/no). Laboratory parameters included: white blood cell count and differential counts, hemoglobin level, erythrocyte sedimentation rate (an acute phase reactant), platelet count, liver enzymes—alanine aminotransferase (ALT) and aspartate aminotransferase (AST), renal function tests—blood urea nitrogen (BUN) and serum creatinine, as well as results of blood and urine cultures.

### 2.2 Outcome Variable Definition

Antibiotic therapy was defined as the administration of any systemic antibiotic agent, including those initiated at the time of hospitalization or prescribed during the inpatient stay. Antibiotics that were used included azithromycin, cefuroxime, ceftriaxone, aminoglycosides, piperacillin/tazobactam, and vancomycin. Indications for antibiotic use, as identified through retrospective medical chart review, were primarily based on clinical factors. These indications included sick‐looking child, radiographic evidence of pneumonic consolidation, clinical suspicion of sepsis, clinical deterioration requiring escalation of respiratory support, and positive blood or urine cultures.

Hospital length of stay (LOS) was defined as the number of days spent in the hospital and categorized as prolonged if equal to or exceeding 5 days. It has been recognized that LOS for RSV‐associated LRI can vary across hospitals and geographical regions due to several factors. Such heterogeneity could be attributed to differences in study populations, including variations in disease severity and the presence of comorbidities. The prolonged hospitalization threshold was set at 5 days based on findings from several studies indicating that this duration approximates the median LOS observed in pediatric RSV‐associated LRI [1].

### 2.3 Statistical Analysis

Statistical analysis was conducted using IBM SPSS Statistics for Windows, version 29 (IBM Corp, Armonk, NY). Normality of continuous variables was assessed using the Shapiro–Wilk test and visual inspection of histograms. Normally distributed variables were described by mean and standard deviation, while nonnormally distributed variables were described by median and interquartile range. Categorical variables were presented as counts and percentages. Differences in categorical variables were analyzed using Pearson's chi‐square (*χ*²) or Fisher's exact test, as appropriate. Cramér's *V* was used to assess the effect size of associations between categorical variables using the following thresholds: weak: > 0.05; moderate: > 0.10; strong: > 0.15; and very strong: > 0.25. To identify factors associated with prolonged hospital stay (≥ 5 days), univariable and multivariable binary logistic regression models were constructed. The model included the following independent variables: age in months (continuous), age less than 12 months (dichotomous), initial peripheral oxygen saturation (SpO₂, continuous), admission respiratory rate (continuous), presence of pneumonia (dichotomous), presence of radiological consolidation (dichotomous), and receipt of antibiotic therapy (dichotomous). The inclusion criteria for variables in the multivariable model were based on clinical relevance and statistical significance in the univariable analysis (*p* < 0.05). Variables with known associations with prolonged hospital stay and those showing significant univariable relationships were considered for inclusion. Odds ratios (ORs) and their corresponding 95% confidence intervals (CIs) were reported for each variable in the multivariable model. A *p*‐value of less than 0.05 was considered statistically significant.

### 2.4 Ethical Approval

The study was approved by the Research Ethics Committee at King Khalid University (HAPO‐06‐B‐001) via approval number ECM#2024‐232, dated 11 February 2024. It was carried out according to the Declaration of Helsinki. Patients’ informed consent was not needed since this was a retrospective observational study without any interventions.

## 3 Results

### 3.1 Antibiotic Use Among the Enrolled Patients

**Figure 1:**
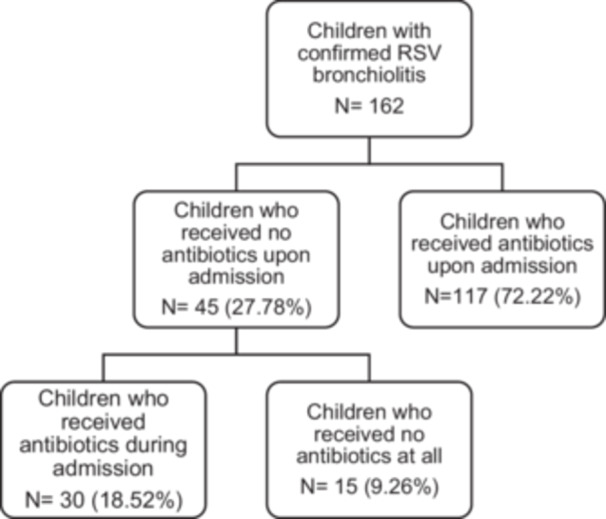
Flowchart showing breakdown of the enrolled patients according to antibiotic use. RSV, respiratory synchial virus.

**Figure 2:**
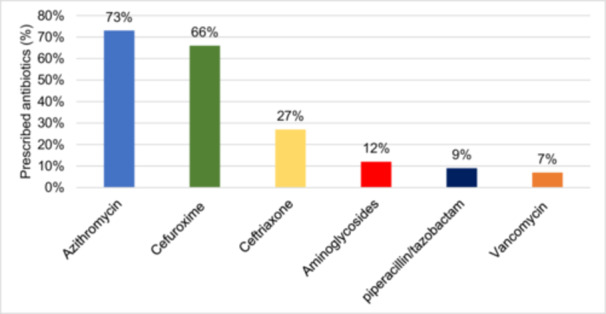
Distribution of antibiotics prescribed for respiratory synchial virus bronchiolitis (total *n* = 147 patients, 117 upon admission, 30 during treatment).

**Figure 3:**
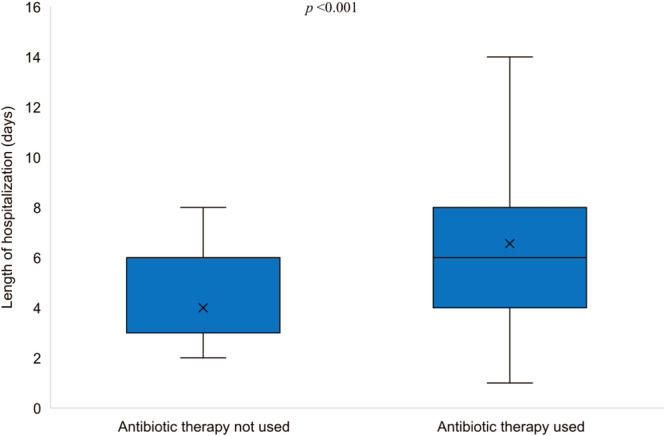
Length of hospitalization for respiratory synchial virus bronchiolitis stratified by antibiotic therapy use.

Figure 1 presents a flowchart breakdown of the enrolled patients according to antibiotic therapy use. Of the 162 patients who were enrolled, 72.22% (*n* = 117) were treated with antibiotics upon admission, while 18.52% (*n* = 30) received antibiotic therapy during hospitalization. Overall, antibiotic therapy was administered to 90.74% (*n* = 147) of the enrolled patients. The most commonly used antibiotics were azithromycin 103 (73.00%), cefuroxime 66.00% (*n* = 97), and ceftriaxone 27.00% (*n* = 40). Less frequently used antibiotics included aminoglycosides 12.00% (*n* = 18), piperacillin/tazobactam 9.00% (*n* = 13), and vancomycin 7.00% (*n* = 10) (Figure 2). The analysis results indicated that patients who received antibiotics had a significantly longer median LOS compared to those who did not (6 vs. 3 days, *p* < 0.001) (Figure 3).

### 3.2 Clinical Characteristics Stratified by Length of Hospitalization

Table 1 shows the demographic and clinical characteristics of the enrolled patients stratified by LOS (< 5 or ≥ 5 days). Children with prolonged LOS exhibited a significantly lower median age compared to those with shorter stays (9 vs. 15 months, *p* = 0.009). No statistically significant differences were observed between LOS groups regarding presenting symptoms or coexisting medical conditions. However, initial median SpO_2_ differed significantly, with children experiencing prolonged LOS exhibiting lower saturation (82% vs. 85%, *p* < 0.001). Children with prolonged LOS had significantly higher respiratory rate at admission than those with hospital stay < 5 days (50 ± 16 and 42 ± 11.9 rate/min, respectively; *p* < 0.001).

**Table 1:** Demographic and clinical characteristics of the enrolled patients stratified by length of hospital stay (< 5 vs. ≥ 5 days).

Variable	Patients with hospitalization < 5 days (*n* = 48, 29.6%)	Patients with hospitalization ≥ 5 days (*n* = 114, 70.4%)	*p*‐value
Age, months, median (IQR)	15 (6–24)	9 (2–18)	0.009
Sex, male, *n* (%)	26 (54.2)	58 (50.9)	0.417
History of contact with sick patient, *n* (%)	45 (93.8)	103 (90.4)	0.642
Onset of symptoms to hospitalization, days, median (IQR)	3 (3–4)	3 (2–4)	0.153
Symptoms, *n* (%)			
– Fever	44 (91.7)	108 (94.7)	0.070
– Wheeze	33 (68.8)	79 (69.3)	0.417
– Cough	47 (97.9)	107 (93.8)	0.646
– Rhinorrhea	40 (83.3)	98 (86.0)	0.226
– Dyspnea	47 (97.9)	108 (94.7)	0.665
– Feeding intolerance	42 (87.5)	104 (91.2)	0.115
Coexisting medical conditions, *n* (%)			
– Premature < 37 weeks GA	16 (33.3)	30 (26.3)	0.279
– Chronic lung disease	4 (8.3)	15 (13.2)	0.255
– Airway disorder	2 (4.2)	3 (2.6)	0.481
– Congenital heart disease	3 (6.3)	15 (13.2)	0.141
– Neurological disorder	3 (6.3)	8 (7.0)	0.558
– Trisomy 21/genetic disorder	0 (0.0)	2 (1.8)	0.483
Vital signs at admission			
– SpO_2_, %, median (IQR)	85 (84–88)	82 (78–86)	< 0.001
– RR,/min, mean ± SD	42 ± 11.9	50 ± 16.0	< 0.001
– HR,/min, mean ± SD	152 ± 22	153 ± 23	0.422
– Body temperature, °C, median (IQR)	38 (37.4–38.5)	38 (37–38.5)	0.582

*Note: p* < 0.05 statistically significant.

Abbreviations: GA, gestational age; HR, heart rate; IQR, interquartile range; m, months; n, number; RR, respiratory rate; SpO_2_, saturation of peripheral oxygen.

### 3.3 Laboratory Findings and Chest Imaging Stratified by Length of Hospitalization

Table 2 summarizes the laboratory and imaging findings of the study participants. Analysis of basic laboratory markers, including hemoglobin, leukocyte count, thrombocyte count, ESR, ALT, and AST, revealed no statistically significant differences (*p* > 0.05) in relation to LOS category. Additionally, no significant differences were observed in the rates of positive blood and urine cultures.

**Table 2:** Laboratory and imaging characteristics of the study population stratified by length of hospital stay (< 5 vs. ≥ 5 days).

Variable	Patients with hospitalization < 5 days (*n* = 48, 29.6%)	Patients with hospitalization ≥ 5 days (*n* = 114, 70.4%)	*p*‐value
WBC (Ref: 4.3–11.0 × 103/μL), mean ± SD	9.2 ± 4.2	8.9 ± 3.4	0.313
ANC (Ref: 1500–8500 cells/μL), mean ± SD	4287 ± 3444	3725 ± 2358	0.122
ALC (Ref: 970–3960/μL), mean ± SD	4080 ± 2281	3938 ± 2415	0.368
Hemoglobin (Ref: 11.5–15.5 g/dL), mean ± SD	12 (11.5–12.8)	12 (11.0–13.3)	0.442
ESR (Ref: 0.0–15 mm/h), mean ± SD	32 (17.0–46.5)	30 (18.5–44.5)	0.714
Platelets, × 1000/mm^3^, mean ± SD	375 ± 165	374 ± 125	0.483
ALT (Ref: 10–35 U/L), median (IQR)	18 (17–27)	19 (15–24)	0.489
AST (Ref: 10–34 U/L), median (IQR)	40 (31–48)	40 (30–49)	0.710
Blood urea nitrogen, mg/dL, median (IQR)	10 (3.8–20)	12 (5.9–16)	0.702
Serum creatinine, mg/dL, median (IQR)	0.24 (0.2–0.3)	0.22 (0.2–0.3)	0.563
Positive blood culture, *n* (%)	4 (8.3)	5 (4.4)	0.273
Positive urine culture, *n* (%)	0 (0.0)	5 (4.4)	0.160
Chest radiographic findings, *n* (%)			
– Normal	6 (12.5)	5 (4.4)	0.072
– Hyperinflation	38 (79.2)	92 (80.7)	0.371
– Atelectasis	19 (39.6)	62 (54.4)	0.043
– Consolidation	11 (22.9)	49 (43.0)	0.008

*Note: p *< 0.05 statistically significant.

Abbreviations: ALC, absolute lymphocyte count; ALT, alanine aminotransferase; ANC, absolute neutrophil count; AST, aspartate aminotransferase; ESR, erythrocyte sedimentation rate; IQR, interquartile range; Ref, reference; SD, standard deviation; WBC, white blood count.

Chest radiographs performed upon admission revealed prevalent radiographic findings among the total study population, including hyperinflation in 80.20% (*n* = 130), atelectasis in 50.00% (*n* = 81), and consolidation in 37.00% (*n* = 60) of patients. To investigate potential associations with prolonged LOS, chi‐square tests of independence were conducted. A statistically significant association between prolonged LOS and atelectasis was observed (*χ*²[1] = 3.562, *p* = 0.043, *V* = 0.151), reflecting a moderate effect size. In addition, a statistically significant association of strong effect size was identified between prolonged LOS and consolidation (*χ*²[1] = 6.443, *p* = 0.008, *V* = 0.203).

### 3.4 Factors Associated With Prolonged Hospitalization ≥ 5 Days

Table 3 presents the univariable and multivariable logistic regression analyses identifying predictors of prolonged LOS. All variables demonstrated significant associations with prolonged LOS in univariable analysis (*p* < 0.05). The multivariable model, incorporating sex and comorbidities, revealed the following adjusted ORs for prolonged LOS, ranked from highest to lowest effect: antibiotic therapy: OR = 7.47 (95% CI: 2.18−25.57), pneumonia: OR = 3.58 (95% CI: 1.67−7.67), age < 12 months: OR = 2.80 (95% CI: 1.32−5.89), consolidation: OR = 2.54 (95% CI: 1.16−5.59), age in months: OR = 0.94 (95% CI: 0.91−0.98) (indicates decreasing odds with increasing age), admission respiratory rate: OR = 1.04 (95% CI: 1.01−1.07), and admission SpO_2_: OR = 0.91 (95% CI: 0.86−0.97) (indicates decreasing odds with higher SpO_2_). These findings suggest that antibiotic therapy, pneumonia, younger age (< 12 months), and presence of consolidation were the strongest independent predictors of prolonged LOS after adjusting for sex and comorbidities. Patients who received antibiotic therapy had 7.47‐times greater odds of experiencing prolonged LOS compared to those who did not receive antibiotics. Similarly, patients with pneumonia, patients under 12 months old, and patients with consolidation had 3.58‐, 2.80‐, and 2.54‐times higher odds of prolonged LOS, respectively. An increase of one unit in respiratory rate was associated with a 1.04‐fold increase in the odds of prolonged LOS. Conversely, every unit increase in SpO_2_ translated to a 0.91‐fold decrease in the odds of prolonged LOS. Similarly, each unit decrease in age in months was associated with a 0.94‐fold decrease in the odds of prolonged LOS.

**Table 3:** Factors associated with prolonged RSV bronchiolitis hospital stay (≥ 5 days) based on multiple logistic regression.

Variable	Univariable			Multivariable		
	OR	95% CI	*p*‐value	aOR	95%CI	*p*‐value
– Age (in months)	0.96	0.93–0.99	0.015	0.94	0.91–0.98	0.002
– Initial SpO_2_	0.91	0.90–0.97	0.003	0.91	0.86–0.97	0.006
– Admission RR	1.0	1.0–1.1	0.005	1.04	1.01–1.07	0.006
Antibiotic therapy						
◦ No	(Ref)	(Ref)	(Ref)	(Ref)	(Ref)	(Ref)
◦ Yes	8.20	2.5–27.2	< 0.001	7.47	2.18–25.57	0.001
Pneumonia						
◦ No	(Ref)	(Ref)	(Ref)	(Ref)	(Ref)	(Ref)
◦ Yes	3.70	1.8–8.0	< 0.001	3.58	1.67–7.67	0.001
Age < 12 months						
◦ No	(Ref)	(Ref)	(Ref)	(Ref)	(Ref)	(Ref)
◦ Yes	2.10	1.1–4.2	0.035	2.80	1.32–5.89	0.007
Consolidation						
◦ No	(Ref)	(Ref)	(Ref)	(Ref)	(Ref)	(Ref)
◦ Yes	2.70	1.2–5.8	0.013	2.54	1.16–5.59	0.020

*Note:* Multivariable logistic regression model adjusted for sex and comorbidities. *p* < 0.05 statistically significant.

Abbreviations: aOR, adjusted odds ratio; CI, confidence interval; OR, odds ratio; RR, respiratory rate; SpO2, saturation of peripheral oxygen.

## 4 Discussion

Despite national and international recommendations advising against antibiotic use in viral respiratory infections, a substantial proportion of patients still receive antibiotics upon admission or during hospitalization [23]. This practice is often driven by legitimate concerns such as suspected bacterial co‐infection, clinically ill children, or the presence of comorbidities [8]. The present study aimed to investigate antibiotic use and predictors of prolonged LOS in children hospitalized with RSV‐LRI. In a retrospective design, 162 patients with RSV‐LRI were included, of whom 90.74% received antibiotic therapy. Notably, 72.22% (117/162) received antibiotics upon admission, while 8.52% (30/162) received them during hospitalization. Additionally, the study revealed a high prevalence of prolonged LOS (≥ 5 days), which occurred for 70.4% of patients. Multivariable analysis identified independent predictors of prolonged LOS as: antibiotic therapy use, presence of pneumonia, consolidation on chest x‐ray, younger age (< 12 months), lower admission SpO_2_, and higher admission respiratory rate.

The observed high prevalence of antibiotic use in this study exceeded expectations, suggesting ongoing challenges in accurately differentiating acute RSV‐LRI from bacterial pneumonia in young children, particularly those under 3 years old. Additionally, the absence of rapid viral diagnostic testing at the time of presentation may further contribute to diagnostic uncertainty and subsequent antibiotic prescribing. Several studies have reported differing percentages of antibiotic use among children less than 3 years with acute RSV‐LRI. Ferrato et al. [24] reported that 52% of infants diagnosed with RSV‐confirmed bronchiolitis received initial treatment with intravenous antibiotics upon admission. However, antibiotic discontinuation following RSV confirmation occurred in 32% of those infants. Our findings are in agreement with those obtained by Akkoc et al. [25], where 100% of children with acute LRI who tested positive for a viral respiratory pathogen received antibiotics (65/65). Furthermore, studies from Israel and Scotland have reported high rates of inappropriate antibiotic prescribing for pediatric LRI, with prevalence reaching 33% and 14%, respectively [17, 19]. Notably, the Scottish study further highlighted that among those inappropriately prescribed antibiotics, 7% of cases involved patients with RSV [17, 19].

An interesting finding of the present study is that patients who received antibiotics had a significantly longer median LOS compared to those who did not (6 vs. 3 days, *p* < 0.001). This finding warrants further investigation to understand if this difference reflects selection bias (i.e., sicker patients receiving antibiotics and having longer stays) or a potential impact of antibiotics on LOS. A comprehensive United States study (*n *= 11,000) evaluating patients under 2 years old with bronchiolitis who required non‐invasive ventilatory support in the ICU found no association between early antibiotic use and improved clinical outcomes [26]. The high rate of antibiotic use in our study, reaching 90.74% (147/162) when considering antibiotics given either upon admission or during hospitalization, likely reflects two key factors. First, concern about potential secondary bacterial infections is a common reason for antibiotic prescribing in such cases. Second, there can be diagnostic uncertainty regarding the viral etiology at initial presentation. Several important questions remain unanswered, including the optimal criteria for initiating antibiotic therapy in children presenting with clinical symptoms suggestive of RSV‐associated LRIs and the appropriate timing for treatment escalation during hospitalization. Further studies addressing these questions need to be undertaken. These findings underscore the complexity of clinical decision‐making in RSV‐associated LRIs, particularly regarding initiation of antibiotic therapy. As clinicians continue to look for strategies to reduce unnecessary antibiotic use and its contribution to antimicrobial resistance, preventive measures become increasingly relevant. The recent availability of RSV maternal vaccines and long‐acting monoclonal antibodies for infants represents a significant advancement in prevention. Their use could reduce the incidence of severe RSV infections requiring hospitalization, and consequently, decrease the inappropriate use of antibiotics often prescribed in viral respiratory illnesses. This has important implications for antimicrobial stewardship and resistance mitigation [27].

Another prominent finding of this work is the high prevalence of prolonged LOS (≥ 5 days), affecting 70.4% of the patients. This is in agreement with prior similar epidemiological studies [20]. In addition, the results provide further evidence that patients with prolonged LOS are characterized by a lower median age compared to those with a shorter hospital stay (9 vs. 15; *p* = 0.009). Furthermore, our data demonstrated that patients with prolonged LOS have significantly lower median SpO_2_ (82% vs. 85%; *p* < 0.001) and higher mean respiratory rate (50 ± 16 vs. 42 ± 11.9; *p* < 0.001). Chest x‐ray findings also showed a higher prevalence of atelectasis and consolidation among patients with prolonged LOS compared to those with a shorter stay (54.4% vs. 39.6%, *p* = 0.043% and 43% vs. 22.9%, *p* = 0.008, respectively). Thus, the independent factors associated with prolonged LOS include antibiotic therapy use, presence of pneumonia, consolidation on chest x‐ray, younger age (< 12 months), lower admission SpO_2_, and higher admission respiratory rate. In accordance with these findings, previous studies have demonstrated a concerning trend of antibiotic overuse in children diagnosed with RSV‐LRIs [17, 28]. The observed association between antibiotic use, pneumonia diagnosis, chest x‐ray consolidation, and prolonged LOS likely reflects disease severity. Patients presenting with these factors are potentially more clinically ill, potentially have co‐existing bacterial pneumonia, and may require longer hospitalization.

Previous studies have reported age less than 12 months to be an independent factor of prolonged LOS [20, 29]. There are two likely causes for the relationship between prolonged LOS and infant age: mechanical and immunological. Mechanically, the smaller caliber of an infant's airways significantly increases airway resistance, as resistance is inversely proportional to the fourth power of the radius. This inherent narrowness is further exacerbated by the inflammatory response characteristic of bronchiolitis, which includes airway edema, mucus production, and epithelial cell necrosis, factors that collectively contribute to airflow obstruction [2, 7, 20]. Immunologically, the innate immune system, the first line of defence against respiratory viruses, is weaker in newborns and particularly in premature infants compared to older children. This immaturity translates to a poorer response to viruses such as RSV, leading to increased susceptibility to severe infections [30, 31].

In our study, lower oxygen saturation (in percentage) and elevated respiratory rate upon admission were independently associated with greater probability of prolonged LOS. Similar to other studies [20], we attribute the association between low oxygen saturation and tachypnea in our study to underlying disease severity, which contributes to the prolonged LOS.

Our inclusion criteria encompassed a broader age range (1–36 months) of patients with RSV LRIs compared to the typical RSV bronchiolitis demographic (infants aged 1–12 months). This may limit generalizability, which should be considered when interpreting the study findings in the context of RSV bronchiolitis. In addition, the absence of a control group in this study design limits our ability to definitively establish the independent effects of the identified predictors on prolonged LOS. A control group would allow for a clearer comparison and isolate the impacts of these factors from other potential confounders. Lastly, the retrospective nature of this study constrains the ability to assess the potential influence of physician experience on the indications of antibiotic initiation. One of the issues that emerges from these findings is the common use of antibiotics among children between 1 and 36 months with LRIs. Further studies elucidating the rationale and necessity for this practice will need to be undertaken. Additionally, implementation of rapid viral screening tests in emergency department settings is recommended to guide more judicious antibiotic use for patients with acute LRIs.

## 5 Conclusion

This study revealed a high rate of antibiotic use among children with RSV‐LRIs, likely influenced by clinical severity and limited access to rapid viral diagnostics at the emergency department. Antibiotic therapy, pneumonia, and younger age were identified as predictors of prolonged hospital stay. These findings support the need for rapid viral testing and further prospective research to guide evidence‐based antibiotic use and improve patient outcomes.

## Author Contributions

Ali Alsuheel Asseri was responsible for the study conceptualization, data management, manuscript drafting, patient recruitment, statistical analysis, data analysis, visualization, and approving the final manuscript for publication.

## Ethics Statement

The study was approved by the Research Ethics Committee at King Khalid University (HAPO‐06‐B‐001) via approval number ECM#2024‐232. It was carried out according to the Declaration of Helsinki.

## Consent

Informed consent was waived because of the retrospective nature of the study and because the analysis used anonymous clinical data.

## Conflicts of Interest

The author declares no conflicts of interest.

## Data Availability

On reasonable request, the corresponding author will provide the datasets used and/or analyzed during the current work.
